# MicroRNA-433 negatively regulates the expression of thymidylate synthase (TYMS) responsible for 5-fluorouracil sensitivity in HeLa cells

**DOI:** 10.1186/1471-2407-13-369

**Published:** 2013-08-02

**Authors:** Keisuke Gotanda, Takeshi Hirota, Nozomi Matsumoto, Ichiro Ieiri

**Affiliations:** 1 Department of Clinical Pharmacokinetics, Graduate School of Pharmaceutical Sciences, Kyushu University, 3-1-1 Maidashi Higashi-ku, Fukuoka 812-8582, Japan

**Keywords:** MicroRNAs, miR-433, Thymidylate synthase, Fluorouracil

## Abstract

**Background:**

Thymidylate synthase (TYMS) is an important folate-dependent enzyme in DNA synthesis and an important target for cancer chemotherapy. High TYMS expression levels in tumors are generally associated with resistance to 5-fluorouracil (5-FU). The cause of the variability in TYMS expression is still not fully understood, however, only a small proportion of the TYMS expression can be explained by *TYMS* genetic polymorphisms. The purpose of this study is to identify novel microRNAs (miRNAs) which regulate the expression of TYMS and to determine whether miRNAs binding to the 3′-untranslated region (UTR) of *TYMS* mRNA affect the proliferation of HeLa cells treated with 5-FU.

**Methods:**

An *in silico* search was performed to find potential binding sites of miRNAs in *TYMS* mRNA. The efficacy of predicted miRNAs at the 3′-UTR of *TYMS* mRNA was evaluated using a dual-luciferase reporter assay. *TYMS* mRNA and protein expression in HeLa cells was quantified with real-time RT-PCR and Western blotting, respectively. The effects of miR-433 on cell proliferative activity were determined by WST-8 assay.

**Results:**

The overexpression of miR-433 was associated with significantly decreased reporter activity in the plasmid containing the 3′-UTR of *TYMS* mRNA (*P* < 0.01). The levels of *TYMS* mRNA and protein in HeLa cells were significantly decreased by the overexpression of miR-433 (*P* < 0.05). Furthermore, miR-433 increased inhibition of cell proliferation in HeLa cells treated with 5-FU at over 2.0 μM.

**Conclusion:**

The results indicate that miR-433 post-transcriptionally regulates the expression of *TYMS* mRNA and protein, and increases sensitivity to 5-FU in HeLa cells. This is the first report showing that a miRNA regulating TYMS expression has a significant impact on sensitivity to 5-FU treatment.

## Background

Thymidylate synthase (TYMS) is an intracellular enzyme critical for *de novo* synthesis of thymidine monophosphate (dTMP), a precursor of the DNA metabolite thymidine triphosphate. Inhibition of TYMS suppresses cellular growth and leads to cell death. Due to this critical function, TYMS has been a major target of anti-cancer drugs for the past 50 years [1]. Five-fluorouracil (5-FU), one of several TYMS inhibitors, is widely used to induce temporary tumor regression and improve survival, especially for gastrointestinal cancers [2]. The level of TYMS expression is known to be related to the response of tumor cells to 5-FU. Overexpression of TYMS in human colon cancer cells induced resistance to fluoropyrimidine [3]. In addition, higher TYMS levels in tumor tissues in cancer patients were associated with resistance to 5-FU-based chemotherapy [4-7].

There is considerable interindividual variability in the clinical response to 5-FU [8]. The 5-FU dose in cancer patients has yet to be well standardized, due to high variability in plasma 5-FU levels, up to 100-fold [9], leading to undesired side effects. This variability may be the major contributor to toxicity and subsequent treatment failure [10,11]. Dose management of 5-FU could therefore prove essential to reducing 5-FU toxicity in patients. Interindividual differences in TYMS expression may be responsible for the different clinical responses to 5-FU. Indeed, a large difference in *TYMS* mRNA expression was observed between colorectal tumor and normal tissues [12]. Three functionally important polymorphisms were identified in the *TYMS* gene: (i) rs34743033: a variable number of 28-bp tandem repeat polymorphisms in the promoter region of the 5′-untranslated enhancer region (UTR) [13]; (ii) rs16430: a 6-bp deletion in the 3′-UTR [14]; and (iii) rs2853542: a G/C polymorphism in the 5′-UTR enhancer region [15]. These polymorphisms showed a significant association with poor outcome in 5-FU-treated patients [16-18]. However, some reports have indicated that *TYMS* polymorphisms did not affect the response to 5-FU therapy [12,19].

MicroRNAs (miRNAs) constitute a class of endogenous, small (19–25 nucleotides), non-coding single-stranded RNAs, and negatively regulate the translation of multiple mRNAs by binding to their 3′-UTRs and inhibiting mRNA translation or breaking down mRNA [20]. It has been reported that miRNAs play an important role as either oncogenes or tumor suppressors, therefore, miRNAs have been increasingly recognized as useful biomarkers, as well as therapeutic tools [21,22]. Moreover, recent evidence suggests that miRNA expression influences chemosensitivity in human cancer cells [23-26].

TYMS expression is reported to be regulated by miR-192 and miR-215 in colorectal cancer cell lines [27]. However, down regulation of TYMS expression by these miRNAs did not sensitize cells to 5-FU in colorectal cancers. Although high TYMS levels are associated with resistance to 5-FU-based chemotherapy [4-7], no miRNAs which repress TYMS expression leading to an increase in sensitivity to 5-FU, have been identified. Interestingly, the 6-bp deletion (rs16430) in the *TYMS* 3′-UTR is reported to decrease mRNA stability *in vitro* and gene expression *in vivo*[28]. These results suggest that certain miRNAs, which target sites proximal to the 6-bp deletion resulting in lower TYMS expression, exist.

In the current study, we identified a novel miRNA regulating the expression of TYMS and evaluated its effect on the proliferation of a human cervical cancer cell line exposed to 5-FU.

## Methods

### Cell culture

The human cervical cancer HeLa cell line was obtained from RIKEN Cell Bank. HeLa cells were cultured in Dulbecco’s modified Eagle’s medium (DMEM) (Sigma-Aldrich, St. Louis, MO, USA) supplemented with 10% fetal bovine serum (FBS) (Nichirei Biosciences Inc., Tokyo, Japan) and incubated at 37°C in 5% carbon dioxide.

### Dual luciferase reporter assays

The full-length 3′-UTR of *TYMS* gene with and without the 6-bp deletion was amplified from human genomic DNA using forward and reverse primers (Forward 5′- TCATCTAGAACCCAGACCTT -3′, Reverse 5′- CCAATCTAGAATACAGCACA -3′). *XbaI* (TaKaRa Inc., Otsu, Japan) restriction sites were created in the primers for cloning. The products were cloned into the pGL3 Promoter vector (Promega, Madison, WI, USA) and named the pGL3 + reference allele or pGL3 + 6 bp deletion allele. Control luciferase constructs were made by ligating oligonucleotides containing a perfect complementary sequence to miR-433 into the *XbaI* site of the pGL3 Promoter vector (pGL3 + miR-433). HeLa cells were plated in a 24-well plate at a density of 6.0 × 10^4^ cells/well and maintained in DMEM with 10% FBS at 37°C in 5% carbon dioxide. After 24 hours, 380 ng of reporter plasmid, 20 ng of pRL-TK (Promega), and negative control precursor miRNA (pre-miR-negative control) (Applied Biosystems Life Technologies, Carlsbad, CA, USA) or pre-miR-433 miRNA precursor (pre-miR-433) (Applied Biosystems) were transfected by using Lipofectamine 2000 (Invitrogen Life Technologies, Carlsbad, CA, USA). Reporter assays were performed at 48 hours post-transfection using the Dual luciferase assay system (Promega), and normalized for transfection efficiency by co-transfected Renilla-luciferase.

### Transfections of miRNA and small interfering RNA (siRNA)

For Western blotting and quantification of mRNA expression, 0.75, 1.5, and 3.0 nM of pre-miR-negative control, pre-miR-433, TYMS siRNA (On-Target plus SMART pool L-004717-00-0005, Dharmacon, Denver, CO, USA) and 1.0 μL/well Lipofectamine 2000 were mixed with Opti-MEM I medium (Invitrogen), then introduced into HeLa cells (1.0 × 10^5^ cells/well) in a 24-well plate.

### RNA isolation and quantitative reverse transcription-PCR analysis of *TYMS* mRNA

Total RNA from HeLa cells was extracted using the miRVana PARIS kit (Ambion Life Technologies, Carlsbad, CA, USA) according to the manufacturer’s instructions at 48 hours after transfection. The RNA samples were then reverse-transcribed into first strand cDNA with 200 ng of total RNA, 2.0 μl of 5 × first strand buffer, 2.0 μl of 0.1 mM DTT, 0.5 μl of 500 μg/ml random primer (Promega), 2.0 μl of 10 mM dNTP mixture and 100 units of SuperScript II RNase H-reverse transcriptase (Invitrogen). The reaction mixture was incubated at 42°C for 60 minutes. The mRNA level was measured with a real-time PCR system (Applied Biosystems). The following primers were used: for the *TYMS* mRNA, 5′-TCTGGAAGGGTGTTTTGGAG-3′ (forward) and 5′-CCTCCACTGGAAGCCATAAA-3′ (reverse), and for the β-actin (*ACTB*) mRNA, 5′-ATGTGGCCGAGGACTTTGATT-3′ (forward) and 5′-AGTGGGGTGGCTTTTAGGATG-3′ (reverse).

### Western blotting

HeLa cells were harvested and homogenized with CelLytic M cell Lysis Reagent (Sigma-Aldrich) and Protease Inhibitor Cocktail (Sigma-Aldrich) at 48 hours after transfection. The lysate samples were separated on 12% SDS-polyacrylamide gels and transferred to polyvinylidene membranes. The membranes were hybridized with a mouse monoclonal antibody against human TYMS (Abcam Inc., Cambridge, MA, USA) and β-actin (Sigma-Aldrich). The immunocomplexes were hybridized with anti-mouse IgG horseradish peroxidase-linked whole antibody (GE Healthcare, Little Chalfont, Buckinghamshire, UK). Membranes were washed two times in PBS-Tween, and specific bands were visualized using the ECL system (GE Healthcare) according to the manufacturer’s instructions.

### 5-FU chemosensitivity

HeLa cells were plated onto a 96-well plate and incubated for 24 hours (4.0 × 10^3^ cells/well). They were then transfected with Pre-miR-negative control (3.0 nM), pre-miR-433 (3.0 nM) or TYMS siRNA (3.0 nM) and treated in 5-FU (Wako, Osaka, Japan) at a final concentration of 0.1, 0.5, 1.0, 2.0, 3.0, 4.0, or 5.0 μM. Cell proliferation was assessed at 120 hours post-transfection using Cell Counting Kit-8 (Dojindo Laboratories Co., Ltd., Kumamoto, Japan) following the manufacturer’s directions.

### Statistical analysis

Statistical analyses were performed with the statistical software program R version 2.13.1 (R Development Core Team, 2011). Means for two groups were compared with an unpaired Student’s *t*-test (two-tailed). Comparisons of means for multiple groups against the pre-miR-negative control transfected cells were analyzed with Dunnett’s multiple comparison tests. A 5% level of probability was considered to be significant.

## Results

### Prediction of miRNAs targeting the 3′-UTR of *TYMS* mRNA

**Figure 1:**
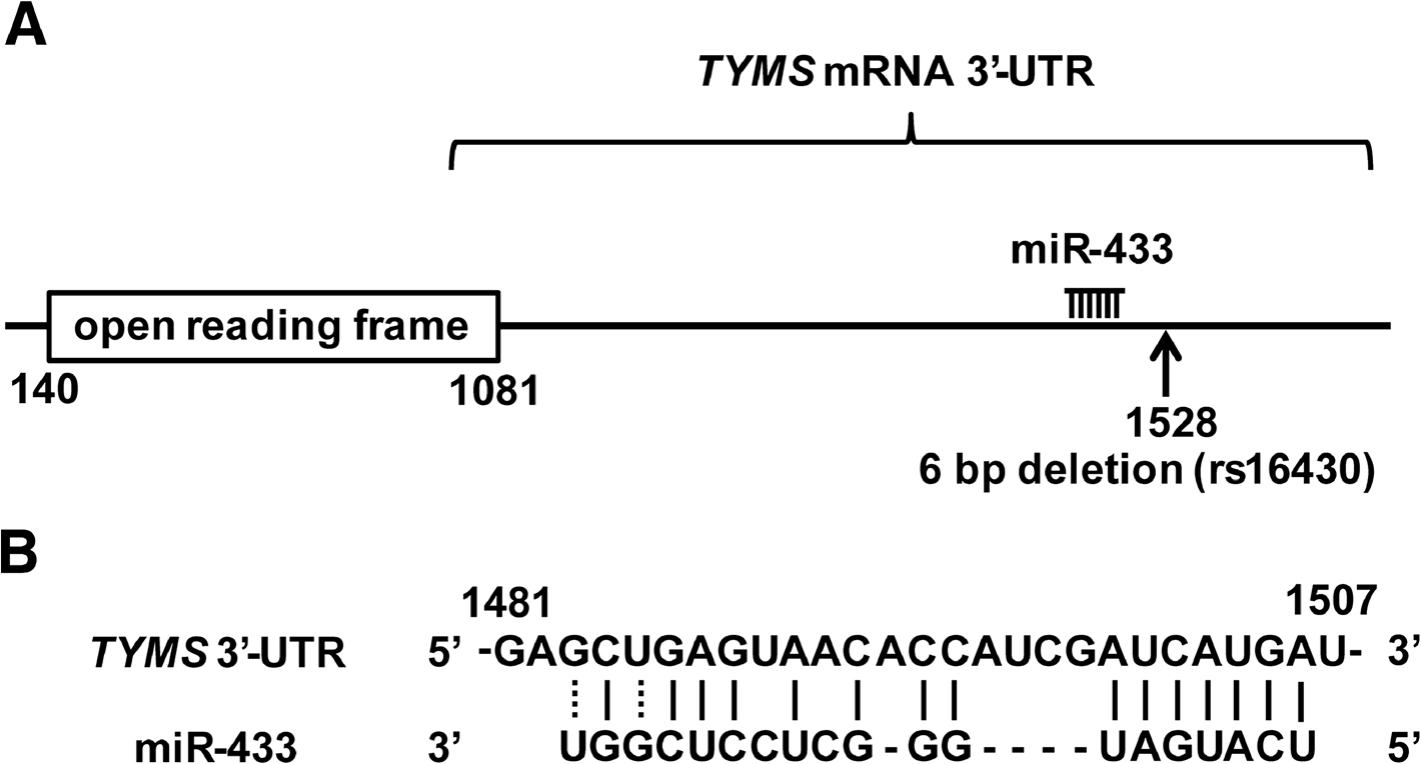
**Prediction of miRNA-binding sites in the 3′-UTR of *TYMS *mRNA. (A)** The locations of the 6-bp deletion (indicated as arrow) and predicted miR-433-binding site in the 3′-UTR of *TYMS* mRNA. **(B)** Homology between the 3′-UTR of *TYMS* mRNA and the predicted target sequence of miR-433. Numbers indicate mRNA positions.

To identify miRNAs targeting the 3′-UTR of *TYMS* mRNA, we used the programs TargetScan (http://www.targetscan.org) and MiRanda (http://www.microrna.org). MiR-433 was selected as a candidate miRNA binding to the 3′-UTR of *TYMS* mRNA based on matching sites (Figure 1). The 6-bp deletion (rs16430) was located 22 bp downstream from the predicted miR-433-binding site.

### Repressive regulation in the 3’-UTR of *TYMS* mRNA by miR-433

**Figure 2:**
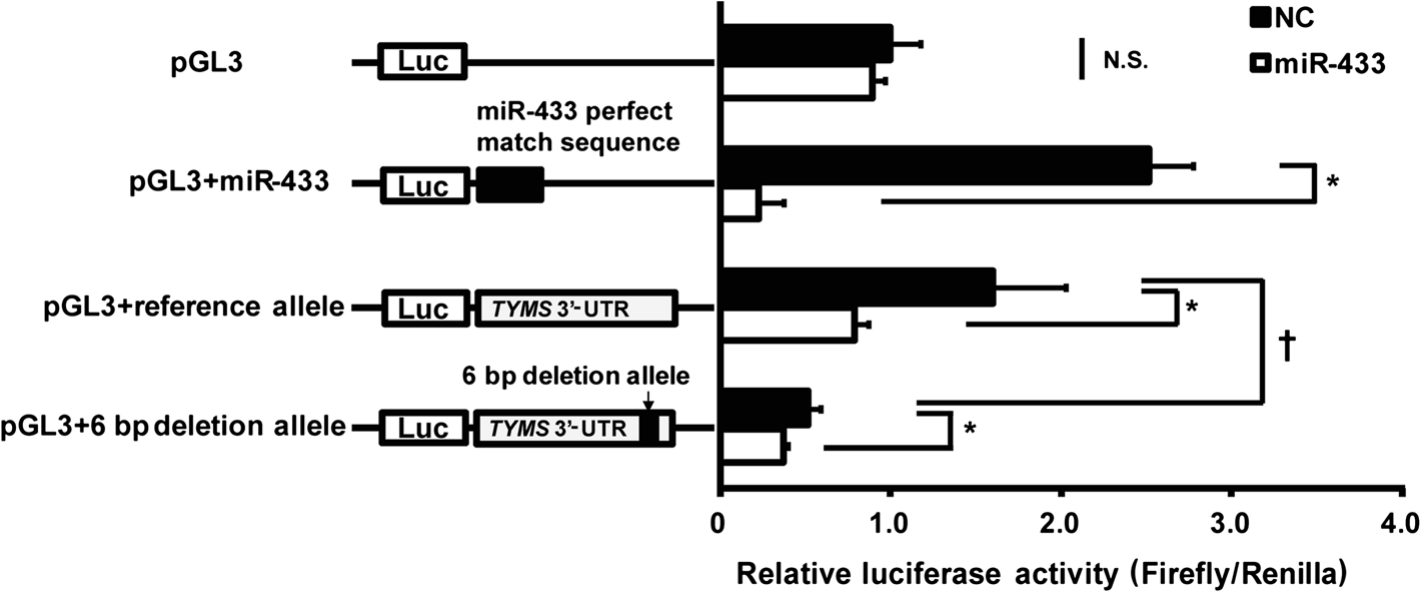
**Repressive regulation of luciferase activity including the human 3′-UTR of *TYMS *by miR-433.** Reporter constructs containing the full-length 3′-UTR of *TYMS* were transiently introduced with 3.0 nM of pre-miR-negative control (NC) or pre-miR-433 (miR-433) into HeLa cells. Luciferase activity was normalized to Renilla luciferase activity. Relative luciferase activity is expressed as a ratio of the Renilla luciferase activity of each precursor. Each column represents the mean ± S.E. for three independent experiments. *, *P* < 0.01, compared with the precursor for the negative control determined by an unpaired two-sample *t*-test. †, *P* < 0.05, compared with pGL3 + 6-bp deletion for the negative control determined by an unpaired two-sample *t*-test. N.S.: not significant.

The sequence complementary to the miR-433-binding site and two full-length human *TYMS* 3′-UTR sequences (the wild type or 6-bp deletion) were inserted downstream of the luciferase reporter gene. We measured luciferase reporter activity as the effects of repressive post-transcriptional regulation by miR-433. The luciferase vector with the 6-bp deletion allele showed significantly lower activity than the wild-type allele in the negative control miRNA-transfected cells (*P* < 0.05, Figure 2). On the other hand, over expression of miR-433 significantly decreased the reporter activity in the cells with the 3′-UTR-inserted vectors compared to the negative control.

### Effects of overexpression of miR-433 on *TYMS* mRNA levels in HeLa cells

**Figure 3:**
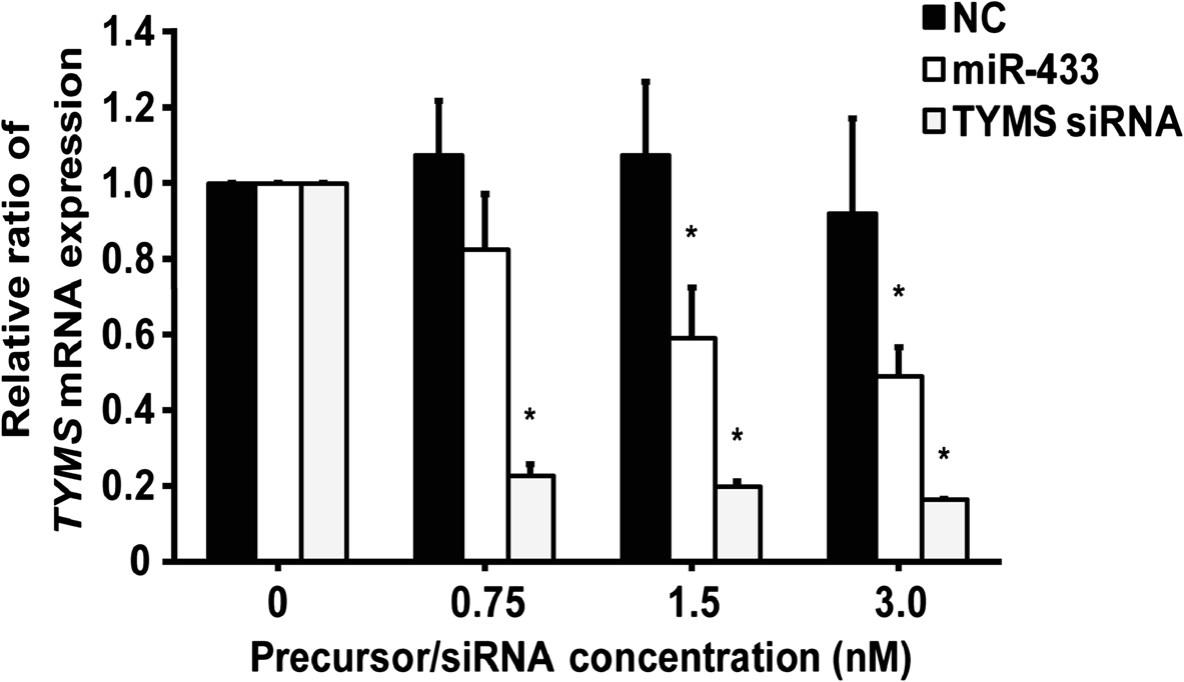
**Effects of overexpression of miR-433 on *TYMS *mRNA levels in HeLa cells.** Hela cells were transfected with pre-miR-negative control (NC), pre-miR-433 (miR-433) or TYMS siRNA (0.75, 1.5 and 3.0 nM). The relative *TYMS* mRNA level was normalized with β-actin. Results represent the mean ± S.D. for four independent experiments. Relative *TYMS* mRNA levels were expressed as ratios of the relative *TYMS* mRNA of the precursor for the negative control. *, *P* < 0.05, compared with the negative control determined by Dunnett’s multiple comparison tests.

To verify the effect of the overexpression of miR-433 on *TYMS* mRNA levels, HeLa cells were temporarily transfected with the pre-miR-negative control, pre-miR-433 or TYMS siRNA (positive control) and *TYMS* mRNA levels were quantitated by real-time RT-PCR analysis using *ACTB* for normalization. The overexpression of miR-433 significantly decreased *TYMS* mRNA levels in a precursor concentration-dependent manner; reduced by approximately 50% at 3.0 nM of pre-miR-433 as compared with the negative control (Figure 3).

### Effects of overexpression of miR-433 on TYMS protein levels in HeLa cells

**Figure 4:**
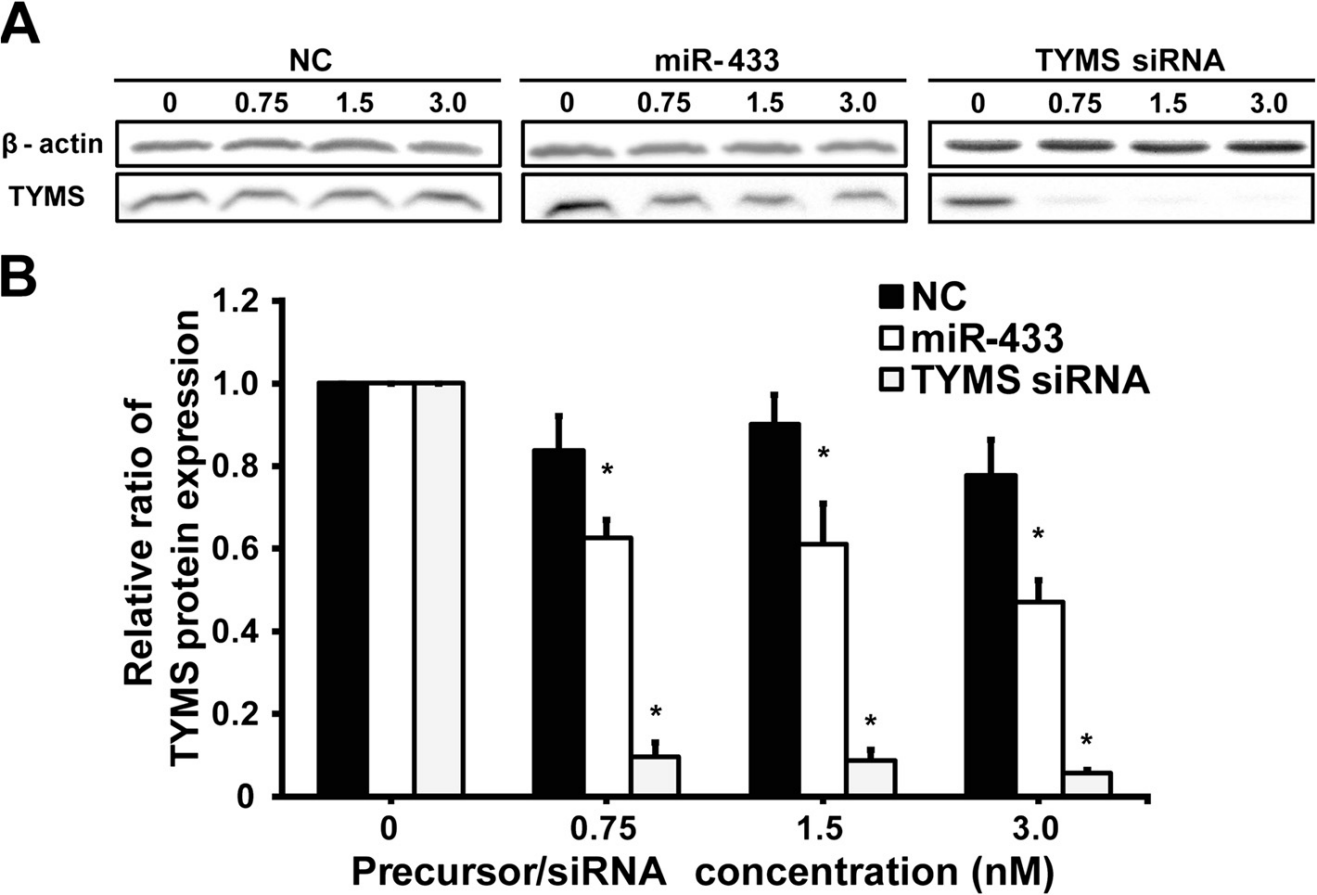
**Effects of overexpression of miR-433 on TYMS protein levels in HeLa cells. (A)** Hela cells were transfected with pre-miR-negative control (NC), pre-miR-433 (miR-433) or TYMS siRNA (0.75, 1.5 and 3.0 nM). TYMS protein levels were determined by Western blotting. **(B)** Relative TYMS protein levels represent the mean ± S.D. for three independent experiments. The TYMS level was normalized with β-actin. Relative TYMS protein levels were expressed as the ratio of the normalized TYMS level to the pre-miR-negative control. *, *P* < 0.05, compared with the negative control determined by Dunnett’s multiple comparison tests.

At all precursor concentrations, siRNA resulted in TYMS protein deficiency, and miR-433 significantly reduced TYMS protein expression compared to the negative control (Figure 4A, B). Transient transfection of 3.0 nM pre-miR-433 resulted in a 40% reduction in TYMS protein expression (Figure 4B).

### Effects of overexpression of miR-433 on sensitivity to 5-FU in HeLa cells

**Figure 5:**
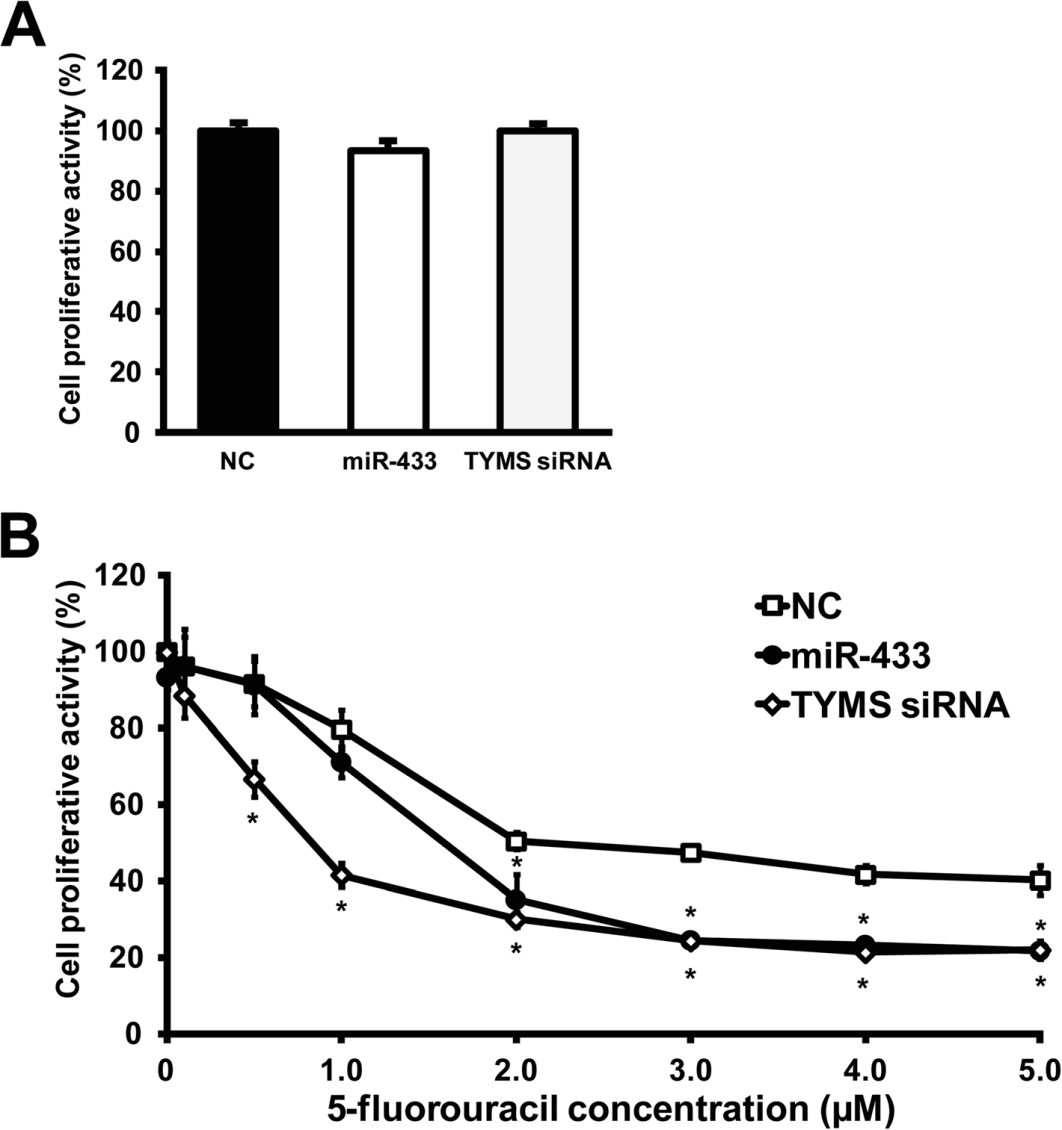
**Effects of miR-433 on the sensitivity to 5-FU in HeLa cells. (A)** HeLa cells were transfected with 3.0 nM of pre-miR-negative control (NC), pre-miR-433 (miR-433) or TYMS siRNA. Cell proliferative activities were determined by WST-8 assay. Each column represents the mean ± S.E. for three independent experiments. **(B)** 5-FU chemosensitivity in HeLa cells was measured by WST-8 assay. Each point represents the mean ± S.D. for three independent experiments. *P < 0.01, compared with the negative control determined by Dunnett’s multiple comparison tests.

To evaluate the effect of miR-433 on the sensitivity to 5-FU treatment, we performed WST-8 assays on HeLa cells transiently transfected with the pre-miR-negative control, pre-miR-433 or TYMS siRNA. First, we analyzed the effects of overexpression of miR-433 and knock-down of TYMS on cell proliferation without 5-FU treatment; there was no change in proliferation among the transfected cells (Figure 5A). TYMS siRNA significantly decreased proliferation in cells treated with over 0.5 μM of 5-FU compared to the pre-miR-negative control (Figure 5B). In cells overexpressing miR-433, proliferation was not affected at 5-FU concentrations ranging from 0.1 to 1.0 μM; however, the decrease in cell proliferation was remarkable over 2.0 μM, and comparable with that by TYMS siRNA over 3.0 μM. These results indicate that overexpression of miR-433 sensitizes HeLa cells to 5-FU treatment.

## Discussion and conclusion

In the present study, we tried to identify a novel miRNA which regulates the expression of TYMS, and evaluate its effects on 5-FU treatment using HeLa cells. After the screening of miRNAs targeting the 3′-UTR of *TYMS* mRNA, miR-433 was predicted to be located in the 3′-UTR proximal to the 6-bp deletion (rs16430). Interestingly, the 6-bp deletion has been associated with decreased mRNA stability and lower gene expression. To demonstrate that miR-433 directly targets the 3′-UTR with or without 6-bp deletion allele, we analyzed the luciferase activity using the vector containing the two alleles. The luciferase vector with the 6-bp deletion allele showed significantly less activity than that with the wild-type vector, suggesting that the 6-bp deletion in the 3′-UTR was involved in the post-transcriptional regulation of TYMS expression. Mandola et al. reported that the 6-bp deletion influenced *TYMS* mRNA stability [28]. As shown in Figure 2, overexpression of miR-433 decreased the luciferase activity, suggesting that miR-433 directly repressed TYMS expression, but it was independently observed for the 6-bp deletion allele. Transfection with the miR-433 precursor significantly decreased *TYMS* mRNA and protein levels in a precursor miRNA concentration-dependent manner (Figures 3 and 4). It was suggested that miR-433 suppresses the expression of the endogenous TYMS protein by controlling the stability of *TYMS* mRNA transcripts. Taking these observations into consideration, miR-433 acts as a repressive regulator, but is not responsible for the decreased mRNA stability due to the 6-bp deletion.

TYMS is considered the primary target of 5-FU [29,30]. It has been suggested that high TYMS expression is correlated with low sensitivity to 5-FU [31,32]. In addition to *TYMS* genetic polymorphisms, the contribution of miRNAs to sensitivity to 5-FU treatment has recently been studied. MiR-192/215 decreased *TYMS* mRNA and protein levels, but significantly reduced sensitivity to 5-FU by targeting cell cycle progression [27]. To date, no miRNA which represses TYMS expression leading to an increase in sensitivity to 5-FU has been identified. In addition to TYMS, miR-433 also targets tumor-associated protein growth factor receptor-bound protein 2 (GRB2) involved in the molecular pathogenesis of gastric cancer [33]. MiR-433 expression was reported to be down-regulated in gastric carcinoma [33]. Furthermore, gastric cancer patients with low expression of miR-433 showed lower survival rates than patients with high expression [34]. Previous reports indicated that miR-433 acts as an anti-oncogenic miRNA (anti-oncomir). However, as shown in Figure 5A, overexpression of miR-433 did not affect cell proliferation in HeLa cells without 5-FU treatment. Our experimental concentration for transfection (at 3.0 nM) was approximately 7-fold lower than that of the previous report (at 25–50 nM) [33]. It is possible that miR-433 does not act as an anti-oncomir at lower concentrations.

Conversely, we showed that miR-433 decreased the proliferation of HeLa cells treated with over 2.0 μM of 5-FU. A concentration of 2.0 μM, approximately 260 μg/L, is close to the clinical target range of 5-FU; indeed, 450–550 μg/L is recommended for FOLFOX6 in metastatic colorectal cancer patients with grade 0–1 toxicity [9]. Hence, miR-433 may improve the efficiency of 5-FU chemotherapy in keeping with Luo’s report [33] that overexpression of miR-433 acts as an anti-oncomir.

This study showed that miR-433 had a significant impact on sensitivity to 5-FU by regulating TYMS expression *in vitro*. Overexpression of TYMS has been linked to drug resistance [7]. Since miR-433 regulates TYMS expression, it is expected to influence the effect of some TYMS-related drugs (*e.g.*, methotrexate). If this is true, miR-433 may have potential as an adjuvant therapeutic to overcome drug resistance.

TYMS, thymidylate synthase; dTMP, thymidine monophosphate; 5-FU, 5-fluorouracil; UTR, untranslated enhancer region; miRNAs, microRNAs.

## Competing interests

The authors declare that they have no competing interests.

## Authors’ contributions

KG: data acquisition, quality control of data, statistical analysis and manuscript preparation; TH: study concepts, design of the study, data analysis, interpretation of the study and manuscript preparation; NM: data acquisition; II: study concepts and manuscript review. All authors read and approved the final manuscript.

## Pre-publication history

The pre-publication history for this paper can be accessed here:

http://www.biomedcentral.com/1471-2407/13/369/prepub
